# CDDO-EA Attenuates Acute Seizures and Suppresses Post-Status Epilepticus Epileptogenesis

**DOI:** 10.3390/antiox15101296

**Published:** 2026-10-07

**Authors:** Yara Sheeni, Prince Kumar Singh, Shweta Maurya, Sereen Sandouka, Tawfeeq Shekh-Ahmad

**Affiliations:** The Institute for Drug Research, The School of Pharmacy, Faculty of Medicine, The Hebrew University of Jerusalem, Jerusalem 91120, Israel

**Keywords:** CDDO-EA, oxidative stress, epileptogenesis, status epilepticus, seizure

## Abstract

We aimed to investigate the acute anticonvulsant and long-term antiepileptogenic effects of the synthetic triterpenoid CDDO-EA in complementary experimental models of epilepsy. CDDO-EA was evaluated in the pentylenetetrazol seizure model and a kainic acid-induced post-status epilepticus model. Acute seizure severity, latency to generalized seizures, oxidative stress and inflammatory markers, spontaneous recurrent seizures, and hippocampal neuronal survival were assessed. In the pentylenetetrazol model, CDDO-EA pretreatment reduced maximal seizure severity and prolonged the latency to stage V-VI seizures. These effects were associated with decreased inflammatory cytokine levels and malondialdehyde levels, alongside increased superoxide dismutase and catalase activities 24 h after pentylenetetrazol administration. In the post-status epilepticus model, two weeks of CDDO-EA treatment significantly delayed the onset of spontaneous recurrent seizures and produced sustained reductions in weekly seizure frequency and cumulative seizure burden. Histological analysis demonstrated greater neuronal preservation in the hippocampal CA1 and CA3 regions of CDDO-EA-treated animals compared with vehicle controls. CDDO-EA exerts both anticonvulsant and disease-modifying effects by attenuating seizure severity, restoring redox-inflammatory homeostasis, preserving hippocampal neurons, and suppressing epileptogenesis after status epilepticus, supporting its therapeutic potential as a novel strategy for epilepsy.

## 1. Introduction

Epilepsy is a chronic neurological disorder characterized by a persistent predisposition to recurrent unprovoked seizures and is frequently accompanied by cognitive, psychiatric, and social consequences that substantially impair quality of life [1]. Despite the availability of numerous antiseizure medications (ASMs), approximately one-third of patients continue to experience seizures after appropriate pharmacological treatment and meet the criteria for drug-resistant epilepsy [1,2]. Current ASMs primarily reduce seizure expression by modulating neuronal excitability, and most fail to prevent epilepsy development or modify the course of disease progression [3,4]. Consequently, therapies that intervene in the mechanisms operating between an initial brain insult and the emergence of spontaneous recurrent seizures remain a major unmet need [3,4].

Acquired temporal lobe epilepsy commonly develops after brain insults such as status epilepticus (SE), traumatic brain injury, stroke, or infection through a prolonged process termed epileptogenesis [3,4]. During this period, excitotoxicity, mitochondrial dysfunction, oxidative stress, neuroinflammation, blood–brain barrier disruption, neuronal loss, and maladaptive network reorganization interact to transform previously nonepileptic tissue into a chronically hyperexcitable network [3,4,5,6]. Oxidative stress is particularly relevant because seizures crucially increase cerebral energy demand and promote reactive oxygen species (ROS) formation through mitochondrial dysfunction and ROS-generating enzymes, including NADPH oxidases [7,8,9]. Previous experimental studies have shown that NOX2 and NOX4 are dynamically regulated after acute seizures and remain elevated in the hippocampus during the post-SE epileptogenic period [9]. We previously showed that targeting NOX2 reduces seizure-associated ROS generation, mitochondrial depolarization, neuronal death, acute seizure burden, and the development or expression of chronic epilepsy, providing causal support for redox dysregulation in epileptogenesis [10,11].

The accumulation of excessive ROS oxidizes membrane lipids, proteins, and nucleic acids, impairs mitochondrial respiration, destabilizes cellular membranes, and amplifies neuronal injury [7,8]. Malondialdehyde (MDA), a product of lipid peroxidation, and 8-hydroxy-2′-deoxyguanosine (8-OHdG), a marker of oxidative DNA damage, are therefore widely used to characterize seizure-associated oxidative injury [7,8,11]. However, endogenous antioxidant enzymes, including superoxide dismutase (SOD) and catalase (CAT), limit oxidative injury by converting superoxide and hydrogen peroxide into less reactive products; however, their capacity may become insufficient during sustained or recurrent seizures [8,12]. Oxidative stress also interacts bidirectionally with neuroinflammation because ROS activate inflammatory signaling, whereas activated glia and inflammatory mediators further increase ROS production and neuronal vulnerability [5,6,11]. Moreover, pro-inflammatory cytokines, including interleukin-1β, interleukin-6, and tumor necrosis factor-α, can enhance glutamatergic transmission, impair inhibitory signaling, disrupt blood–brain barrier integrity, and lower seizure threshold, whereas anti-inflammatory mediators such as interleukin-10 can restrain these responses [5,6,11,13]. These reciprocal processes create a self-perpetuating pathological cycle that is unlikely to be adequately controlled by strategies directed only at ion channels or a single terminal ROS [6,11,13].

Nuclear factor erythroid 2-related factor 2 (Nrf2) is a central transcriptional regulator of cellular redox homeostasis, mitochondrial support, detoxification, and inflammation [12,14]. Under basal conditions, Nrf2 is retained by Kelch-like ECH-associated protein 1 (Keap1) and targeted for ubiquitin-dependent degradation, whereas oxidative or electrophilic stress stabilizes Nrf2, promotes its nuclear translocation, and induces antioxidant response element-dependent genes [12,14]. The Nrf2 transcriptional program includes NAD(P)H:quinone oxidoreductase 1, heme oxygenase-1, glutamate-cysteine ligase, glutathione-associated enzymes, and other cytoprotective systems that collectively enhance antioxidant capacity rather than merely scavenging individual radicals [12,14]. Previous evidence supports this pathway in epilepsy, as Nrf2-deficient mice exhibit greater kainate-induced seizure severity, hippocampal injury, and mortality, whereas Nrf2 activation is observed in experimental and human temporal lobe epilepsy [15,16,17]. In rat seizure models, Nrf2 activation is temporally and regionally regulated and is prominently expressed in hippocampal neurons after both acute seizures and SE [18,19]. Pharmacological Keap1 inhibition, combined redox therapy, dimethyl fumarate treatment, and seizure-responsive Nrf2 gene therapy have each reduced neuronal injury, seizure burden, and epilepsy development in preclinical models, establishing Nrf2 as a credible disease-modifying target [20,21,22,23].

CDDO-EA, CDDO-ethyl amide or RTA405, is a synthetic oleanane triterpenoid developed as a potent pharmacological activator of the Keap1-Nrf2 pathway [24,25,26]. CDDO-EA increases Nrf2-dependent transcription when Keap1 regulation remains functional and has demonstrated antioxidant and anti-inflammatory activities in several experimental disease settings [27]. Importantly for neurological application, CDDO-EA has shown biological activity in the brain after systemic administration [25,28,29,30]. CDDO-EA and related triterpenoids activate Nrf2/antioxidant response element signaling, induce antioxidant and mitochondrial-supportive genes, and prolong survival in a mouse model of amyotrophic lateral sclerosis [25]. Systemic synthetic triterpenoids also suppress inflammatory cytokine programs and improve disease outcomes in experimental autoimmune encephalomyelitis [28]. In cerebral ischemia, post-insult CDDO-EA increases Nrf2 and heme oxygenase-1 expression, promotes a reparative microglial/macrophage phenotype, and reduces neurological injury [29]. CDDO-EA also increased cerebral Nrf2 signaling, reduced inflammatory and vascular injury, preserved blood–brain barrier integrity, and improved survival in experimental cerebral malaria [30]. These observations indicate that CDDO-EA can engage coordinated antioxidant, anti-inflammatory, mitochondrial, and neurovascular defenses; however, whether it can suppress acute seizures or modify post-SE epileptogenesis has not been established [24,25,27,28,29,30].

In the present study, we investigated the therapeutic potential of CDDO-EA in complementary acute seizure and post-status epilepticus models of epilepsy. We first determined whether CDDO-EA pretreatment can alter pentylenetetrazol-induced seizure susceptibility and whether its acute effects were accompanied by modulation of lipid peroxidation, endogenous antioxidant enzyme activity, and pro-inflammatory cytokine levels. We then examined its antiepileptogenic potential by initiating CDDO-EA treatment after kainic acid-induced status epilepticus and continuously monitored the development of spontaneous recurrent seizures using wireless electrocorticography. Finally, we assessed long-term hippocampal oxidative DNA damage and inflammatory gene expression to determine whether early CDDO-EA intervention produced sustained effects on the redox-inflammatory environment associated with the development of epilepsy. Through this integrated experimental approach, we sought to distinguish the acute anticonvulsant effects of CDDO-EA from its potential to modify post-SE epileptogenesis and establish its therapeutic relevance as a redox- and inflammation-directed strategy for epilepsy.

## 2. Materials and Methods

### 2.1. Animals and Ethical Approval

Male Sprague–Dawley rats (150–200 g; Hebrew University strain, originally obtained from Harlan, Jerusalem, Israel) were used in this study. Animals were housed individually under controlled environmental conditions (23 °C, 50–60% relative humidity, and a 12-h light/dark cycle) with ad libitum access to food and water. Rats were acclimatized to the animal facility for 3 days (acute PTZ experiments) or 7 days (post-SE experiments) before experimentation. All experimental procedures were conducted in accordance with the guidelines of the Association for Assessment and Accreditation of Laboratory Animal Care International and were approved by the Institutional Animal Care and Use Committee of the Hebrew University of Jerusalem (Approval No. MD-20-16254-5).

### 2.2. Experimental Design, Animal Grouping, and Drug Administration

The study comprised two experimental cohorts designed to evaluate the acute anticonvulsant effects and post-status epilepticus antiepileptogenic efficacy of CDDO-EA.

For acute seizure cohort I, 18 naïve male Sprague–Dawley rats were randomly assigned to either the CDDO-EA-treated PTZ group (*n* = 9) or the vehicle-treated PTZ group (*n* = 9). Animals in the treatment group received freshly prepared CDDO-EA (RTA405; 30 mg/kg, i.p.), a dose selected based on previous studies demonstrating biological activity of CDDO-EA at doses ranging from 15–60 mg/kg/day, as well as our previous experience with the compound. The selected dose was well tolerated in our experimental conditions, with no signs of overt toxicity observed. CDDO-EA was formulated in a vehicle containing 20% polyethylene glycol 400 (PEG 400) and 80% 0.5% methylcellulose (MC; Sigma-Aldrich, St. Louis, MO, USA), whereas control animals received an equivalent volume of vehicle. One hour after treatment, animals were subjected to a two-dose subcutaneous pentylenetetrazol (scPTZ) paradigm. Behavioral seizure activity was monitored continuously for 2 h following the first PTZ injection. Twenty-four hours after seizure induction, the animals were sacrificed, and brain tissue samples were collected for biochemical analyses.

For post-status epilepticus cohort-II, 25 naïve male Sprague–Dawley rats underwent implantation of wireless ECoG transmitters and were subsequently subjected to kainic acid-induced status epilepticus. Following the exclusions described in Section 2.5 Induction of status epilepticus, 16 animals were randomly assigned to either the CDDO-EA-treated group (*n* = 8) or the vehicle-treated group (*n* = 8). Animals received CDDO-EA (30 mg/kg/day, i.p.) or an equivalent volume of vehicle once daily for 14 consecutive days, beginning 1 h after diazepam-mediated termination of SE. Continuous 24/7 wireless video-ECoG monitoring was performed for 11 weeks to assess the development and progression of spontaneous recurrent seizures. At the end of the monitoring period, animals were euthanized, and brain tissue samples were collected for biochemical and immunohistochemical analyses.

### 2.3. Subcutaneous Pentylenetetrazol (scPTZ) Seizure Model

Acute generalized seizures were induced using a two-dose subcutaneous pentylenetetrazol (scPTZ) paradigm as described previously [10]. Rats were administered CDDO-EA (RTA405; 30 mg/kg, i.p.) or an equivalent volume of vehicle. One hour later, animals received an initial subcutaneous injection of PTZ (50 mg/kg; Sigma-Aldrich, St. Louis, MO, USA), followed by a second injection (30 mg/kg, s.c.) 30 min after the first dose. Animals were continuously observed for behavioral seizure activity throughout the 2-h monitoring period. Behavioral seizure severity was monitored according to the modified Racine scale [31], and seizure incidence, latency to the first observable seizure, seizure duration, and highest seizure stage reached by each animal were recorded.

### 2.4. Animal Surgery: Implantation of ECoG Transmitter

Animals assigned to post-status epilepticus cohort-II underwent implantation of wireless electrocorticography (ECoG) transmitters, as previously described [11]. All naïve animals were anesthetized with 3% isoflurane (Terrell™, USP; Piramal Critical Care, Bethlehem, PA, USA) and maintained at 1.8–2.3% throughout surgery. Animals were positioned in a stereotaxic frame (Kopf Instruments, Tujunga, CA, USA) and received preoperative meloxicam (1 mg/kg, s.c.; Chanelle Pharma, Loughrea, Co. Galway, Ireland) and buprenorphine (0.3 mg/kg, s.c.;VetViva Richter GmbH, Wels, Austria). A single-channel wireless ECoG transmitter (A3028E; 512-Hz sampling rate; Open-Source Instruments Inc., Rochester, NH, USA) was implanted in the subcutaneous pocket. Two electrode wires were positioned subdurally through cranial burr holes using coordinates relative to the bregma. The recording electrode was placed over the right hippocampal region (AP: −2.8 mm; ML: +2.5 mm), and the reference electrode was positioned over the contralateral hemisphere (AP: −6.0 mm; ML: +3.5 mm). The electrodes were stabilized with skull screws and tissue adhesive (VetBond Tissue Adhesive; 3M, Saint Paul, MN, USA), and the electrode assembly was secured using dental cement (Simplex Rapid; Kemdent, Swindon, Wiltshire, UK). At the completion of surgery, the animals received 3–5 mL of warmed Ringer’s solution and amoxicillin (100 mg/kg; Betamox LA; Norbrook Laboratories Limited, Newry, Co. Down, Northern Ireland, UK) and were returned to their home cages. A 7-day postoperative recovery period was allowed before the initiation of status epilepticus induction and continuous 24/7 ECoG monitoring.

### 2.5. Induction of Status Epilepticus

Status epilepticus (SE) was induced by a single intraperitoneal injection of kainic acid (KA; 10 mg/kg; Hello Bio, Bristol, UK), freshly prepared at 5 mg/mL in sterile 0.9% saline. Following KA administration, animals were continuously monitored, and convulsive seizure severity was scored according to the modified Racine scale [31,32]. SE onset was defined as the development of sustained class V or recurrent class IV–V motor seizures, characterized by rearing, forelimb clonus, and loss of postural control. Seizure activity was allowed to continue for 2 h from the onset of SE, after which diazepam (10 mg/kg, i.p.; Assival^®^, Teva Pharmaceutical Industries Ltd., Kfar Saba, Israel) was administered to terminate convulsions. Post-SE supportive care included meloxicam (1 mg/kg, s.c.) once daily for the first 3 days. Animals were regularly monitored for body weight changes, general recovery, hydration, mobility, and signs of pain or distress.

Of the 25 animals subjected to KA administration, five failed to meet the predefined SE criterion, three died during or after SE, and one was excluded because of transmitter failure and inadequate ECoG signal quality. The remaining 16 animals were randomly allocated to either the vehicle-treated group or the CDDO-EA-treated group, with eight animals per group. Treatment with CDDO-EA (30 mg/kg/day, i.p.) or an equivalent volume of the vehicle was initiated 1 h after diazepam administration and continued once daily for 14 consecutive days. Animals remained under continuous 24/7 wireless video-ECoG monitoring throughout the treatment and subsequent epileptogenesis period to assess the development and progression of spontaneous recurrent seizures.

### 2.6. ECoG Data Acquisition and Seizure Analysis

Animals in cohort-II were housed individually and continuously monitored using wireless video-electrocorticography (vECoG) throughout the recording period. ECoG signals were acquired and stored using the Neuroarchiver software (https://www.opensourceinstruments.com/Electronics/A3018/Neuroarchiver.html, accessed on 28 August 2026, Open-Source Instruments Inc.). The recordings were segmented into 4-s epochs and screened using six signal features: total power, intermittency, coastline, coherence, asymmetry, and spectral power within the 12–30 Hz band. Each feature was normalized to a 0–1 range and compared with a reference library containing manually validated seizures from at least three animals. Epochs with a Euclidean distance of <0.2 from a reference seizure pattern were flagged as candidate events using the automated detection algorithm described by Open-Source Instruments. All candidate events were subsequently reviewed by an investigator blinded to treatment allocation. Electrographic seizures were defined as rhythmic, high-amplitude discharges exceeding twice the baseline amplitude, showing temporal evolution in frequency and/or amplitude, followed by postictal suppression. A subset of the detected events was further verified against synchronized video recordings obtained using externally positioned CCTV cameras (Microseven). A second investigator, blinded to the treatment allocation, reviewed the synchronized videos to confirm the behavioral correlates of electrographic seizures.

### 2.7. Quantitative Real-Time PCR

Total RNA was isolated from brain tissues using TRI Reagent (Sigma-Aldrich, St. Louis, MO, USA). RNA concentration and purity were determined using a NanoDrop OneC spectrophotometer (Thermo Scientific, Waltham, MA, USA). Complementary DNA was synthesized from 1 μg of total RNA using oligo(dT)15 primers and the GoScript™ Reverse Transcription System (Promega, Madison, WI, USA). Quantitative real-time PCR was performed using a CFX Connect Real-Time PCR Detection System (Bio-Rad, Hercules, CA, USA). Each 15 μL reaction contained 7.5 μL PerfeCTa SYBR Green FastMix (Quantabio, Beverly, MA, USA), 3 μL primer mixture at a final concentration of 500 nM per primer, 1.5 μL DEPC-treated water (Sigma-Aldrich, USA), and 3 μL cDNA template. The amplification protocol consisted of an initial denaturation at 95 °C for 10 min, followed by 40 cycles at 95 °C for 5 s and 60 °C for 15 s, with final steps at 65 °C for 5 s and 95 °C for 30 s. All reactions were performed in duplicate, and primer sequences were adapted from our previous report [10] as listed in Table 1: The relative target gene level was normalized to GAPDH and calculated using the method of Pfaffl et al. [33]. All the results were expressed relative to the corresponding vehicle-treated control group.

### 2.8. Assessment for Oxidative Stress Markers

#### 2.8.1. Determination of Lipid Peroxidation

Malondialdehyde (MDA) levels were measured in brain homogenates using the thiobarbituric acid reactive substances (TBARS) assay, as described previously [34]. Tissue homogenates were mixed with thiobarbituric acid reagent and incubated at 95 °C for 60 min to allow formation of the MDA-TBA adduct. After cooling, the samples were centrifuged, and the resulting supernatants were transferred to a 96-well plate. Absorbance was measured at 532 nm, and MDA concentrations were calculated from a standard curve and expressed as nmol/mg tissue.

#### 2.8.2. Assessment of Superoxide Dismutase Activity

Superoxide dismutase (SOD) activity was quantified using a colorimetric assay kit (Cayman Chemical, Ann Arbor, MI, USA, Item No. 706002) according to the manufacturer’s instructions. The assay measures the inhibition of tetrazolium salt reduction by superoxide radicals generated through the xanthine oxidase reaction. Absorbance was recorded at 450 nm, and SOD activity was determined from the standard curve and expressed as units per milliliter. One unit of SOD activity was defined as the amount of enzyme required to achieve 50% dismutation of the superoxide radicals.

#### 2.8.3. Assessment of Catalase (CAT) Activity

Catalase (CAT) activity was quantified using a colorimetric assay kit (Abcam, Cambridge, UK, ab83464) following the manufacturer’s instructions. Tissue samples were homogenized in ice-cold assay buffer, centrifuged at 10,000× *g* for 15 min at 4 °C, and the resulting supernatants were used for analysis. Catalase-mediated decomposition of H_2_O_2_ was allowed to proceed for 30 min at 25 °C, after which the remaining H_2_O_2_ was detected using the OxiRed™ developer system. Absorbance was measured at 570 nm, and CAT activity was calculated from the H_2_O_2_ standard curve and expressed as mU/mL. One unit of catalase activity was defined as the amount of enzyme required to decompose 1 μmol of H_2_O_2_ per minute at 25 °C.

### 2.9. Statistical Analysis

Statistical analyses were performed using GraphPad Prism version 9.3.1 (GraphPad Software, USA). Data acquisition and analysis were conducted by investigators blinded to treatment allocation. Quantitative data are presented as mean ± standard error of the mean (SEM), with n representing the number of individual animals or independent biological samples, as specified in the corresponding figure legends. Comparisons between two groups were performed using an unpaired Student’s *t*-test, whereas datasets involving more than two groups or multiple experimental factors were analyzed using one-way or two-way analysis of variance (ANOVA), followed by Tukey’s or Dunnett’s post hoc test, as appropriate. The incidence of seizures in the acute PTZ cohort was analyzed using Fisher’s exact test. Longitudinal seizure frequency data from the post-SE cohort were analyzed using a generalized log-linear mixed-effects model incorporating animals as a random effect with an autoregressive covariance structure and treatment group, week, and their interaction as fixed effects. Statistical significance was set at *p* < 0.05. Group sizes were based on prior experience with variability in comparable experimental paradigms, and the number of animals included in each analysis is reported in the corresponding figure legends.

## 3. Results

### 3.1. CDDO-EA Pretreatment Reduces PTZ-Induced Seizure Severity and Delays the Onset of Convulsive Seizures

**Figure 1:**
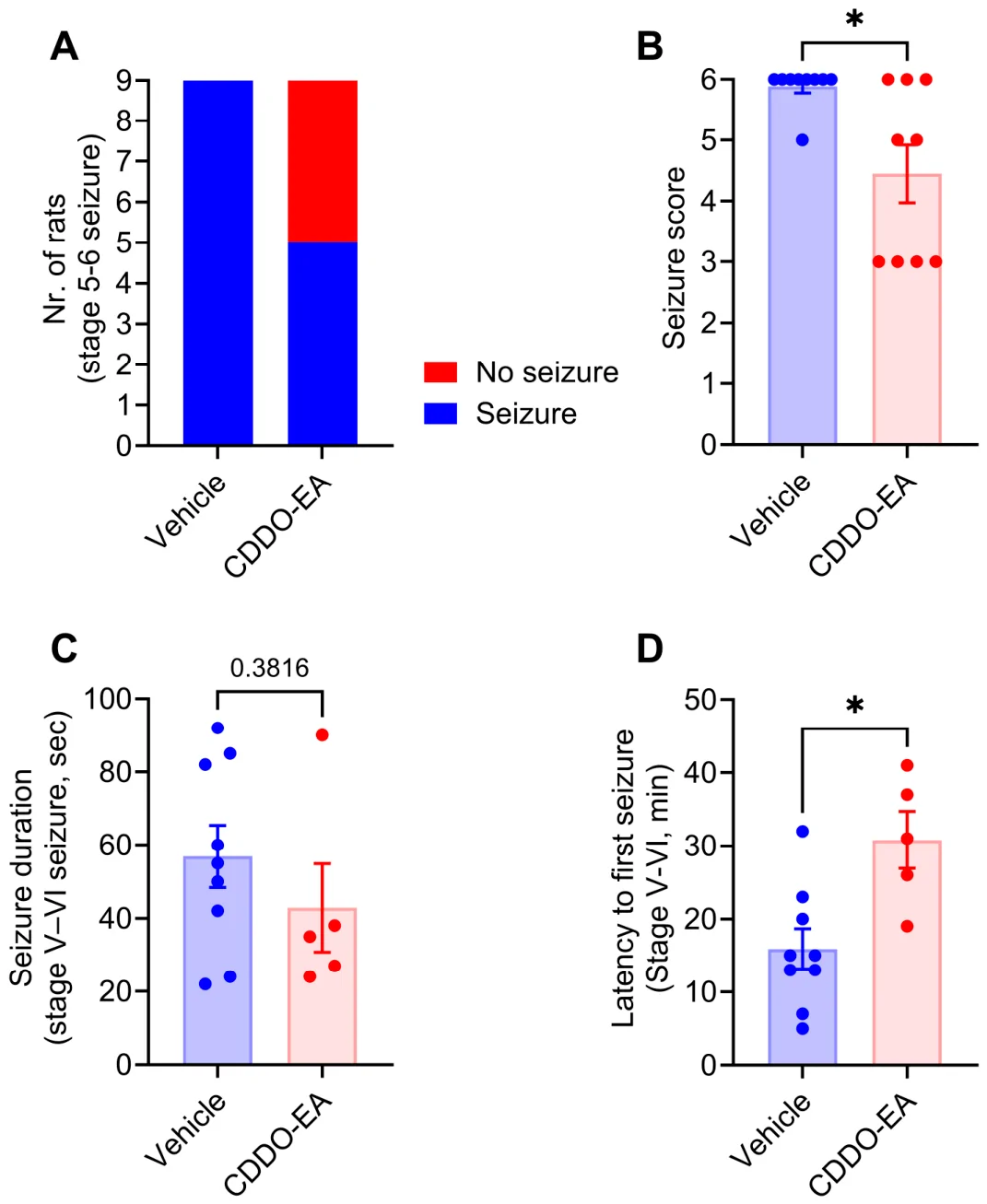
**CDDO-EA pretreatment attenuates seizure severity and delays the onset of PTZ-induced convulsive seizures.** (**A**) Number of animals that developed stage V–VI convulsive seizures during the 2-h observation period. (**B**) Maximum seizure severity experienced by each animal. (**C**) Duration of stage V–VI convulsive seizures among animals that developed stage V–VI seizures. (**D**) Latency to the first stage V–VI seizure onset in both vehicle- and CDDO-EA-pretreated animal cohort. *n* = 9 animals per group. Data are presented as mean ± s.e.m. Statistical significance was determined either by fisher’s exact test (**B**) or two-tailed Mann–Whitney tests (**B**–**D**). * *p* < 0.01.

The acute anticonvulsant effect of CDDO-EA was evaluated using the two-dose scPTZ seizure model following pretreatment with CDDO-EA or vehicle. All animals developed stage V-VI convulsive seizures following pretreatment with vehicle (9/9), whereas the incidence of stage V-VI convulsive seizures decreased from 100% to 55.6% in CDDO-EA-pretreated animals (9/9 vs. 5/9, vehicle vs. CDDO-EA cohort, Fisher’s exact test, *p* = 0.0824; Figure 1A). CDDO-EA pretreatment significantly reduced the maximum seizure severity experienced by each animal during the observation period (Mann–Whitney U = 16, exact two-tailed, *p* = 0.0226; Figure 1B). Among animals that developed stage V-VI seizures, the median convulsive seizure duration was 55 s in vehicle-treated animals and 35 s in CDDO-EA-treated animals; however, this difference was not statistically significant (Mann–Whitney *U* = 15.5, exact two-tailed *p* = 0.3816; Figure 1C). In contrast, CDDO-EA significantly prolonged the latency to the first stage V-VI seizure from a median of 15 min in the vehicle group to 31 min in the CDDO-EA group (Mann–Whitney *U* = 5, exact two-tailed *p* = 0.0180; Figure 1D). Together, these findings demonstrate that CDDO-EA pretreatment reduced PTZ-induced seizure severity and delayed progression to stage V-VI convulsive seizures, although it did not significantly alter the incidence or duration of convulsive seizure.

### 3.2. CDDO-EA Pretreatment Suppresses Pro-Inflammatory Cytokine Expression Following PTZ-Induced Seizures

**Figure 2:**
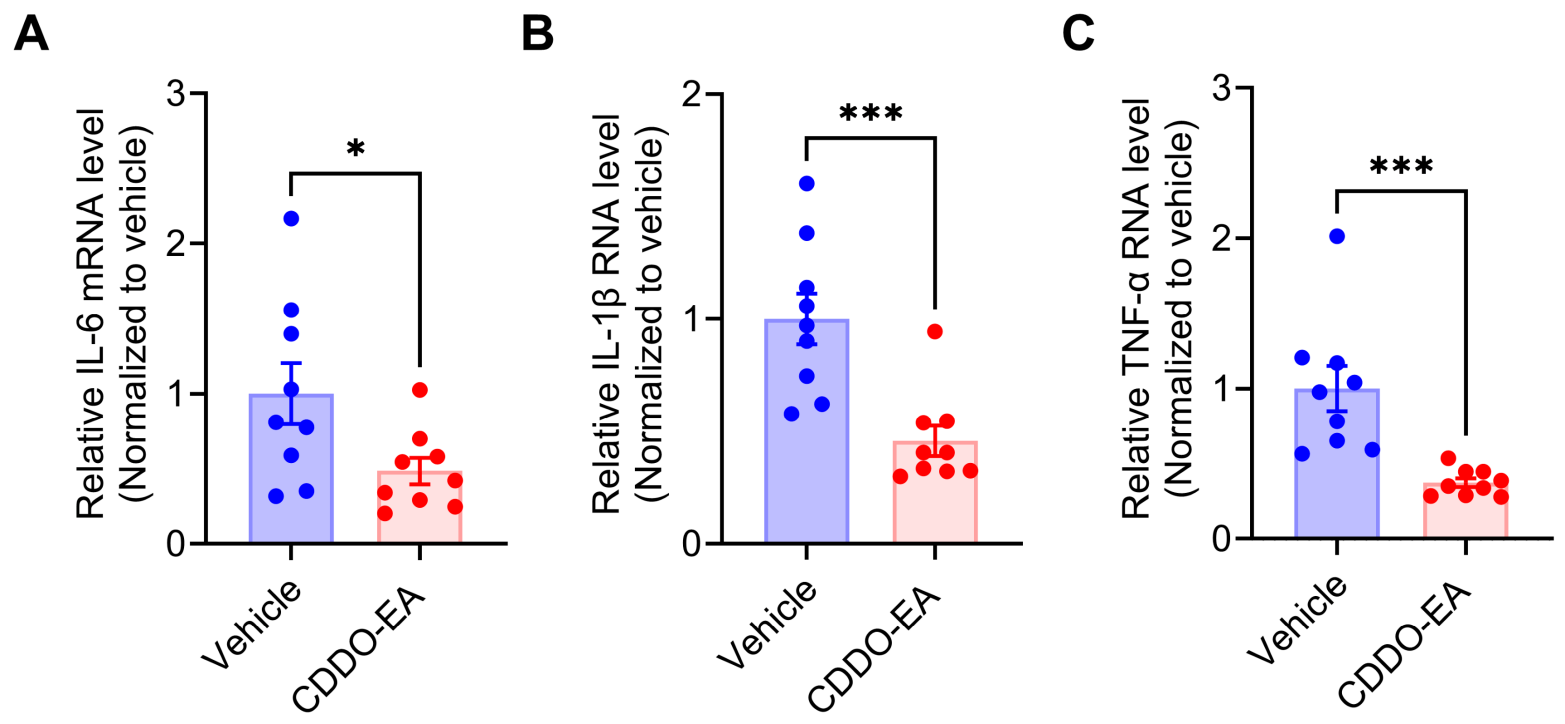
**Effects of CDDO-EA on inflammatory cytokine levels following PTZ-induced seizure.** Relative mRNA levels of IL-6 (**A**), IL-1 β (**B**), and TNF-α (**C**) in the brain of vehicle- and CDDO-EA-pretreated animals following 24 h post-PTZ administration. *n* = 9 animals per group. Data are presented as mean ± s.e.m. Statistical significance was determined by an unpaired *t*-test. * *p* < 0.05, *** *p* < 0.001.

To determine whether the acute anticonvulsant effects of CDDO-EA were accompanied by changes in neuroinflammatory signaling, pro-inflammatory cytokine levels were assessed in whole-brain tissue 24 h after PTZ administration. CDDO-EA pretreatment significantly reduced IL-6 mRNA level compared to that in vehicle-treated animals (*p* = 0.0278; Figure 2A). Similarly, IL-1β and TNF-α levels were significantly reduced in CDDO-EA-treated animals compared to vehicle-treated animals (*p* = 0.0008, and 0.0009; Figure 2B and Figure 2C, respectively). These findings demonstrate that CDDO-EA pretreatment attenuated the acute pro-inflammatory response associated with PTZ-induced seizures.

### 3.3. CDDO-EA Pretreatment Attenuates Oxidative Stress Following PTZ-Induced Seizures

**Figure 3:**
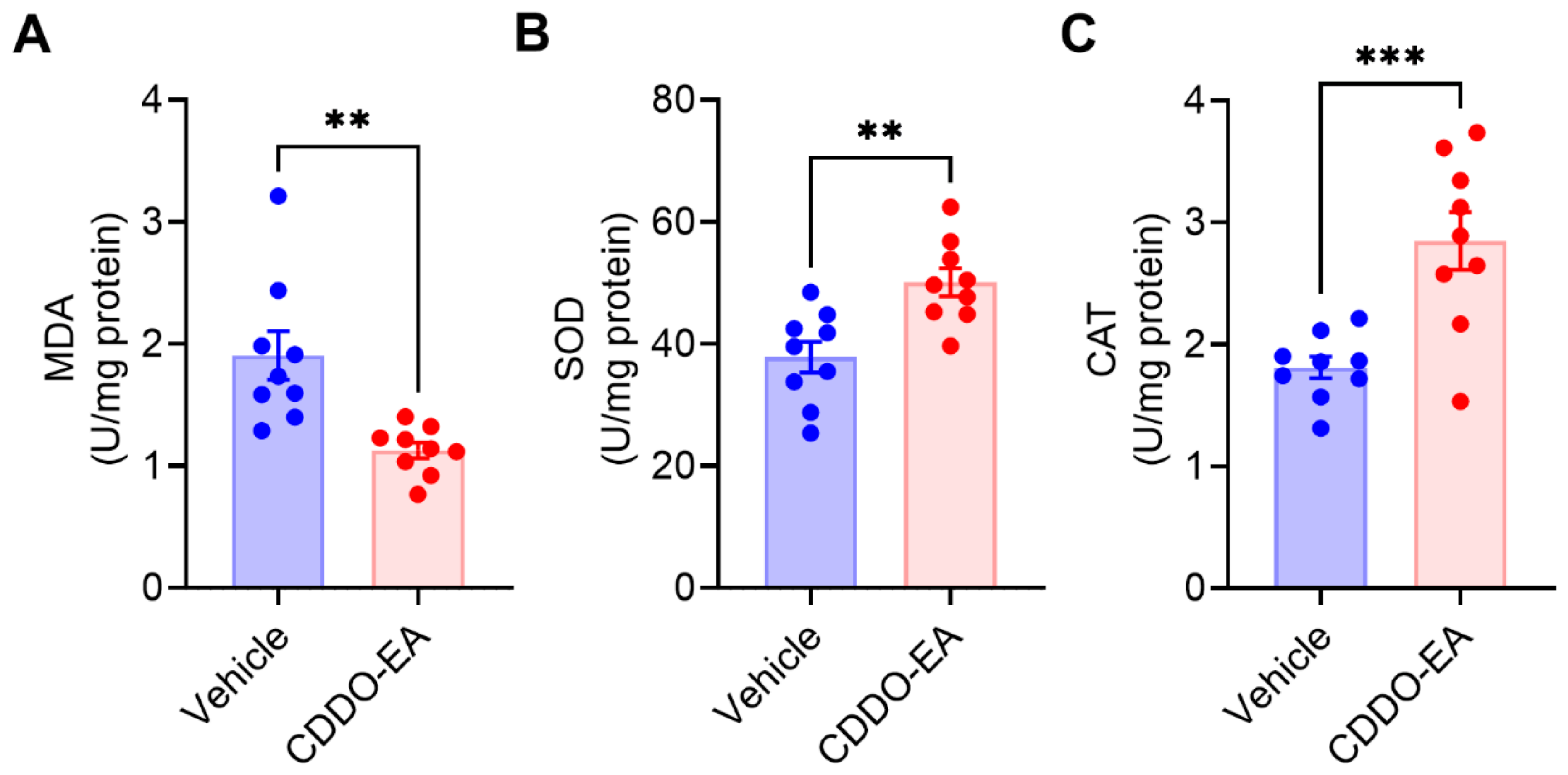
**Effects of CDDO-EA pretreatment on oxidative stress markers following PTZ-induced seizures.** Malondialdehyde (MDA) levels (**A**), superoxide dismutase (SOD) activity (**B**), and catalase (CAT) activity (**C**) were measured in the brain of CDDO-EA- and vehicle-pretreated animals following 24 h post-PTZ administration. Data are presented as mean ± SEM. *n* = 9 animals per group. Statistical significance was determined by unpaired *t*-test. ** *p* < 0.01, *** *p* < 0.001.

To determine whether the anticonvulsant and anti-inflammatory effects of CDDO-EA were associated with modulation of oxidative stress, lipid peroxidation and antioxidant enzyme activities were assessed in whole-brain homogenates 24 h after PTZ administration. CDDO-EA pretreatment significantly reduced MDA levels compared to vehicle-treated animals (*p* = 0.0019; Figure 3A), indicating decreased lipid peroxidation. In parallel, superoxide dismutase activity was significantly increased in the CDDO-EA-treated group (*p* = 0.0025; Figure 3B). Moreover, we also observed that CDDO-EA significantly enhanced catalase activity compared to the vehicle group (*p* < 0.001; Figure 3C) 24 h after the PTZ-induced seizure. Together, these findings indicate that CDDO-EA pretreatment limits PTZ-associated oxidative damage while enhancing endogenous antioxidant defenses.

### 3.4. CDDO-EA Therapy Suppresses Epileptogenesis and Reduces Spontaneous Seizure Burden Following Status Epilepticus

**Figure 4:**
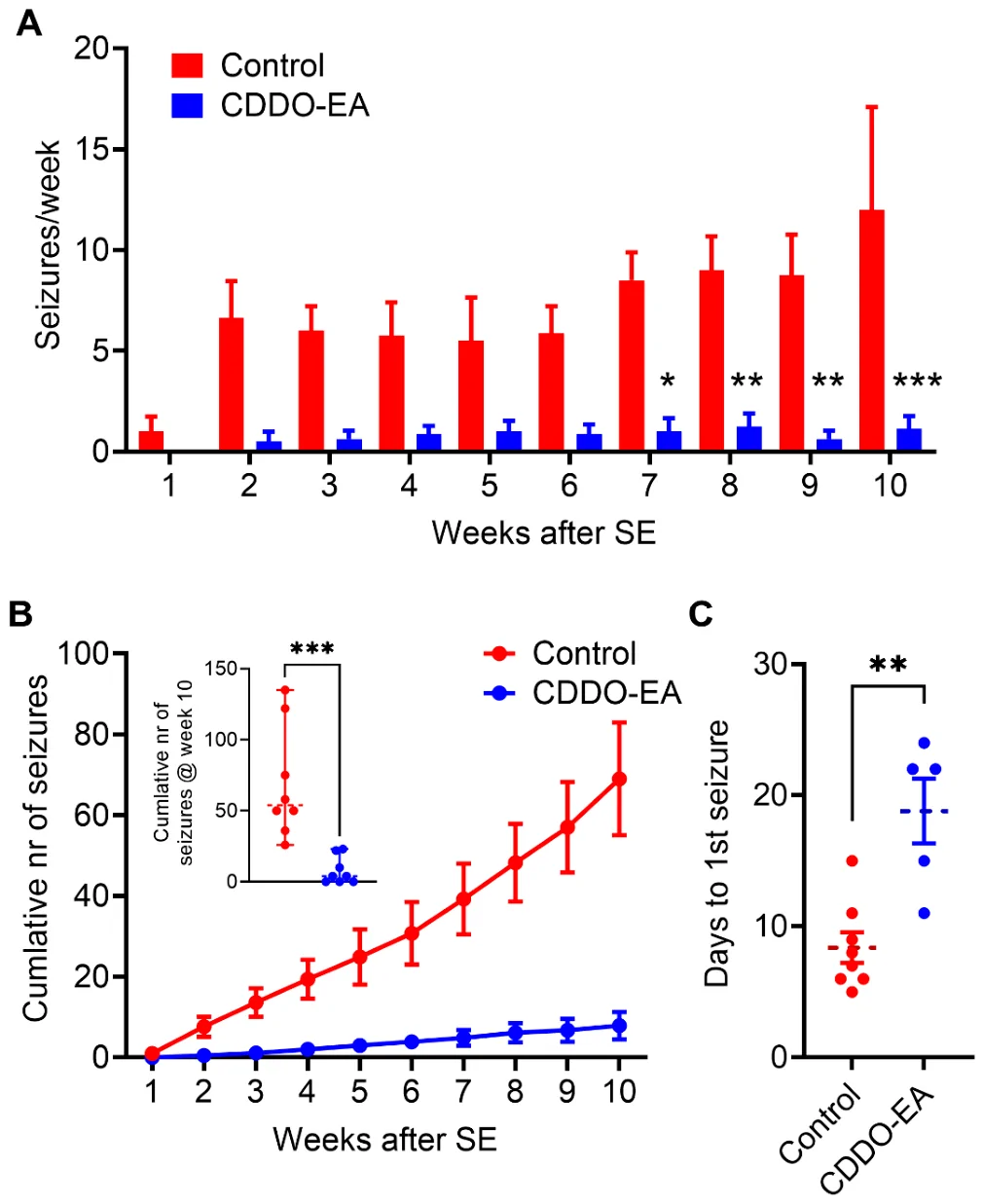
**CDDO-EA therapy suppresses epilepsy development and spontaneous recurrent seizure burden following status epilepticus.** (**A**) Weekly seizure frequency in control (red) and CDDO-EA (blue) treated animal cohorts over a 10-week duration following SE. (**B**) Cumulative number of spontaneous seizures recorded over the monitoring period. The inset shows the total cumulative number of seizures at week 10 experienced by individual animals. (**C**) Latency to the first spontaneous recurrent seizure after status epilepticus. *n* = 8 animals per group. Data are presented as mean ± s.e.m. Statistical significance was determined by a generalized log-linear mixed model, incorporating the random effect of the animal (autoregressive covariance) and fixed effects of treatment group, time, and the interaction between treatment group and time (panel (**A**)); unpaired *t*-test (panel (**B**) inset and (**C**)). * *p* < 0.05, ** *p* < 0.01, *** *p* < 0.001.

Next, we determined whether early intervention immediately after status epilepticus could modify the subsequent development of epilepsy. We induced SE, and following one-hour post-SE termination with diazepam, animals received either CDDO-EA or vehicle once daily for two weeks. All animals underwent continuous 24/7 wireless video-ECoG monitoring to record spontaneous recurrent seizure activity over 10 weeks. Control animals exhibited a progressive increase in weekly seizure frequency over the 10-week monitoring period, with the highest seizure burden observed during the later stages of epileptogenesis (Figure 4A,B). In contrast, CDDO-EA-treated animals exhibited a lower seizure frequency throughout the study, with significant reductions relative to controls at weeks 7–10 (*p* = 0.0122 at week 7, *p* = 0.0084 at week 8, *p* = 0.0048 at week 9, and *p* < 0.0001 at week 10; Figure 4A). This sustained separation between the groups indicates that the effects of the 2-week CDDO-EA treatment extended well beyond the treatment period. Consistent with the weekly seizure profile, the cumulative number of spontaneous seizures increased steadily in the control animals but remained substantially lower in the CDDO-EA-treated group (Figure 4B). At week 10, the total cumulative seizure burden was significantly reduced by CDDO-EA compared to that by vehicle treatment (*p* < 0.001; Figure 4B, inset). Interestingly, CDDO-EA therapy also significantly prolonged the latency to the first spontaneous recurrent seizure following status epilepticus (*p* < 0.01; Figure 4C).

Together, these findings demonstrate that early intervention with CDDO-EA delayed the onset of spontaneous seizures, reduced the chronic seizure burden, and suppressed the progressive increase in spontaneous recurrent seizure frequency following SE, supporting a sustained antiepileptogenic effect.

### 3.5. CDDO-EA Therapy Does Not Produce Sustained Changes in Inflammatory Cytokine Levels

**Figure 5:**
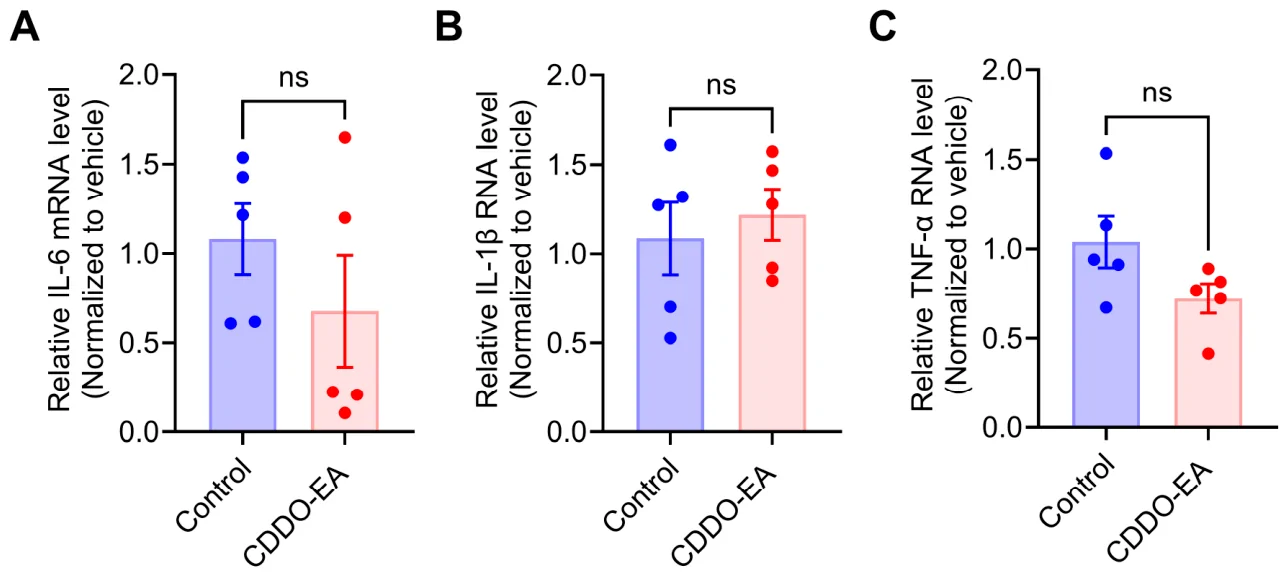
**Effects of CDDO-EA therapy on inflammatory cytokines following spontaneous recurrent seizure activity.** Relative mRNA levels of IL-6 (**A**), IL-1β (**B**), and TNF-α (**C**) in the brain tissue from control and CDDO-EA-treated animal cohorts after 10 weeks of vECoG monitoring. *n* = 5 animals per group. Data are presented as mean ± s.e.m.; *n* = 5 animals per group. Statistical significance was determined by an unpaired *t*-test. ns, not significant.

We next examined whether the early 14-day CDDO-EA treatment produced persistent changes in brain inflammatory signaling after 10 weeks of vECoG monitoring. However, we did not observe any significant differences in IL-6, IL-1β, and TNF-α mRNA levels in the control and CDDO-EA-treated animals (Figure 5A–C). Although IL-6 and TNF-α levels tended to be lower in the CDDO-EA-treated group, these differences were not statistically significant.

### 3.6. CDDO-EA Therapy Preserves Hippocampal Neuronal Density Following Spontaneous Recurrent Seizures

**Figure 6:**
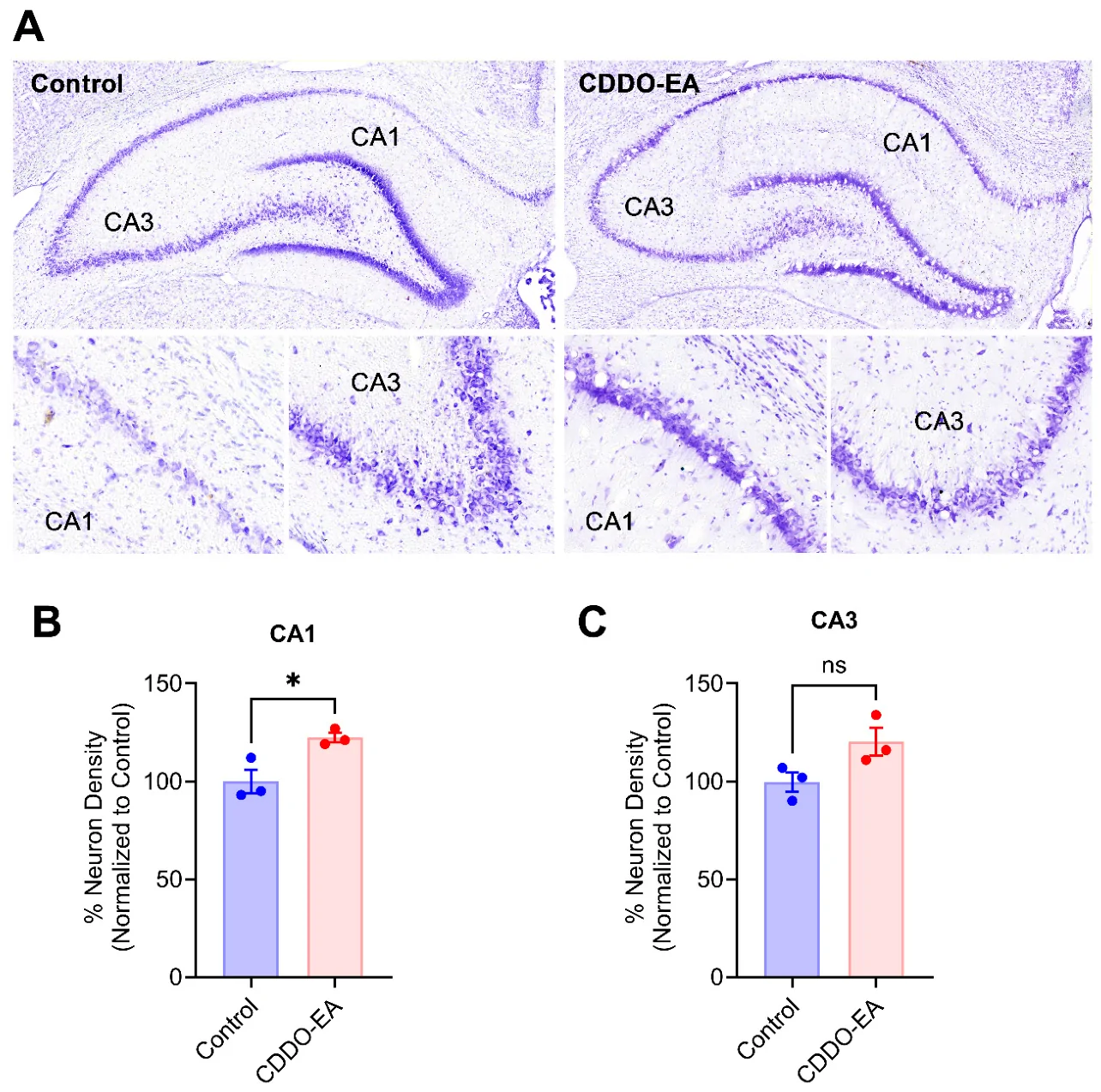
**CDDO-EA therapy protects hippocampal neurons following spontaneous recurrent seizure activity.** (**A**) Representative Nissl-stained hippocampal sections from control and CDDO-EA-treated animals after 10 weeks of vECoG monitoring. Higher-magnification images of the CA1 and CA3 subfields are shown in the lower panels. (**B**,**C**) Quantification of neuronal density in the CA1 (**B**) and CA3 (**C**) regions, expressed as a percentage of the control group. Data are presented as mean ± SEM; *n* = 3 animals per group. Statistical significance was determined using an unpaired *t*-test. * *p* < 0.01; ns, not significant.

Next, we examined whether early CDDO-EA treatment after SE attenuated hippocampal neuronal loss associated with recurrent spontaneous seizures. We quantified neuronal density by Nissl staining in both control and CDDO-EA-treated animals after 10 weeks of vECoG monitoring (Figure 6A). Compared with control-treated animals, the CDDO-EA-treated cohort exhibited significantly higher neuronal density in the CA1 region (*p* < 0.01; Figure 6B), whereas no significant difference was observed in the CA3 region (Figure 6C). These findings suggest that early CDDO-EA treatment protects hippocampal neurons, particularly in the CA1 region, from neuronal injury associated with recurrent spontaneous seizures.

## 4. Discussion

Epileptogenesis is sustained by interacting oxidative, inflammatory, metabolic, and neurovascular disturbances that continue after the initial insult and progressively increase the probability of spontaneous seizures [35,36,37]. This creates a therapeutic rationale for interventions that strengthen endogenous cytoprotective networks rather than suppressing neuronal excitability alone. CDDO-EA is particularly relevant in this context because it can activate Keap1-Nrf2 signaling and regulate inflammatory responses; however, its effects on acute seizure susceptibility and post-SE epilepsy development have not been previously established [22,23,24,25,27,28]. Our findings indicate that CDDO-EA acts at both stages, as it limits the progression to severe convulsive activity in the acute PTZ model and produces a persistent reduction in spontaneous seizure development when administered during the early post-SE period.

In the scPTZ model, CDDO-EA significantly reduced the maximum seizure score and prolonged the latency to stage V–VI seizures (Figure 1). The incidence of stage V–VI convulsions also decreased from 100% to 55.6%, although this difference did not reach statistical significance, and seizure duration was unchanged among animals that developed stage V–VI seizures. This pattern suggests that CDDO-EA primarily increases resistance to seizure progression rather than uniformly preventing PTZ responsiveness or terminating established convulsive episodes. A similar anticonvulsant profile was previously observed with the Hsp90 inhibitor, cemdomespib, which reduced PTZ-induced seizure susceptibility and severity and delayed seizure onset, although it also shortened seizure duration, an effect not observed with CDDO-EA [38]. Cemdomespib pretreatment reduced seizure susceptibility and maximum severity and prolonged seizure latency, supporting the concept that pharmacological regulation of cellular stress pathways can limit progression to generalized convulsive activity [38]. However, cemdomespib also shortened seizure duration, whereas CDDO-EA did not significantly alter the duration of stage V–VI seizures among animals that convulsed. This difference suggests that the two compounds may share an ability to increase resistance to seizure recurrence while differing in their influence on already established convulsive activity. Such an effect is consistent with the contribution of redox imbalance to seizure initiation and propagation. Previously, kainate, kindling, and post-SE models demonstrated that seizures lead to lipid oxidation, mitochondrial DNA damage, impaired DNA repair, persistent mitochondrial redox dysfunction, and ROS-dependent respiratory deficits [35,36,37,39,40,41,42]. Moreover, rapid NMDA receptor-dependent activation of NADPH oxidase and hydrogen peroxide production have been directly implicated in seizure initiation [43]. By limiting these redox-sensitive processes, CDDO-EA may reduce the recruitment of neuronal networks required for the progression of generalized motor seizures. However, because acute Nrf2 target engagement was not measured, the behavioral protection cannot be attributed exclusively to the transcriptional activation of Nrf2.

The changes in oxidative stress markers provide biological support for our interpretation. CDDO-EA reduced MDA levels while increasing SOD and CAT activities 24 h after PTZ administration (Figure 3). These enzymes are crucial as SOD converts superoxide to hydrogen peroxide, whereas CAT removes hydrogen peroxide, thereby limiting hydroxyl radical formation and membrane lipid oxidation. The concurrent increase in both activities is therefore more informative than the alteration of either enzyme alone, suggesting the modulation of endogenous antioxidant capacity. Previous studies have similarly linked antioxidant interventions to reduced seizure severity or seizure-associated injury. A catalytic antioxidant preserved mitochondrial function, hippocampal neurons, and cognition after SE, although it did not modify spontaneous seizure development, illustrating that neuroprotection does not necessarily imply antiepileptogenesis [44]. In the post-SE model, α-tocopherol showed attenuation of lipid peroxidation, glial activation, and hippocampal degeneration [45], whereas ferulic acid attenuated PTZ kindling and oxidative injury [46]. More recent studies reported protection against kainate-induced seizures through Nrf2-associated regulation of ferroptosis by quercetin and activation of PI3K/Akt/Nrf2 signaling by biochanin A [47]. These findings support the biological relevance of the antioxidant response observed here while emphasizing that the relationship between oxidative injury and seizure modification depends on the molecular target, timing, and duration of treatment.

CDDO-EA also suppressed IL-6, IL-1β, and TNF-α expression following PTZ-induced seizures (Figure 2). These cytokines can actively modulate seizure susceptibility rather than merely serving as markers of tissue injury. IL-1β enhances NMDA receptor-mediated calcium influx through Src-family kinase signaling and can rapidly exacerbate seizures through phosphorylation of the NR2B receptor subunit [48,49,50]. TNF-α has receptor-dependent actions, including regulation of seizure susceptibility through p75 receptors and modification of excitatory synaptic strength through glial TNF-α-dependent synaptic scaling [51,52]. Innate immune signaling through the HMGB1-TLR4 pathway can likewise facilitate acute and recurrent seizures [48]. The pathological relevance of immune-mediated excitotoxicity is further supported by evidence that glutamate receptor–reactive IgG from patients with intractable epilepsy damaged neural cells and induced recurrent generalized seizures and neuronal loss in naïve rats [53]. The reduction in all three cytokines therefore provides a plausible inflammatory component to the delayed convulsive progression produced by CDDO-EA. This interpretation is also consistent with studies of the related triterpenoid CDDO-Me, which reduced post-SE microglial activation, monocyte infiltration, NF-κB signaling, reactive astrogliosis, and astroglial injury [54,55]. Nevertheless, because molecular measurements were obtained only after PTZ exposure, the present design cannot determine whether cytokine suppression was a direct pharmacological effect or partly secondary to the lower seizure severity in CDDO-EA-treated animals.

The most important finding is the long-term effect of early CDDO-EA therapy following SE. We confirmed that control animals exhibit the expected progressive increase in spontaneous seizure frequency, whereas CDDO-EA-treated animals maintained substantially lower seizure activity, showed a reduced cumulative burden, and experienced a longer delay before the first spontaneous seizure (Figure 4). Progressive seizure accumulation after kainate-induced SE is well documented and reflects an epileptogenic process that continues beyond the emergence of the first spontaneous seizure [56]. Additionally, our approach to using verified spontaneous seizures as the primary endpoint is crucial, as fast-ripple occurrence can arise from coincident neuronal firing and varies with network synchrony and vigilance state, limiting its specificity as a standalone marker of epileptogenic activity. The sustained separation between groups is particularly relevant because CDDO-EA was administered for only 14 days, whereas seizure monitoring continued for several weeks after treatment ended. Thus, the findings are more consistent with modification of early epileptogenic processes than with continuous symptomatic seizure suppression. However, CDDO-EA-treated animals also exhibited higher neuronal density in the CA1 regions (Figure 6), indicating that the reduced chronic seizure burden was accompanied by preservation of hippocampal neurons. In contrast, IL-6, IL-1β, and TNF-α levels did not differ between groups at week 10 (Figure 5), possibly because treatment ended eight weeks before tissue collection; this late measurement may not capture transient anti-inflammatory effects occurring during the early post-SE period. The importance of an early therapeutic window is also supported by neuron-specific WWOX gene replacement, which suppressed epileptiform activity and produced durable, dose-dependent rescue of neuroinflammation and neurological dysfunction in WWOX-related epileptic encephalopathy [57]. Consistent with these findings, a 2-week course of cemdomespib after KA-induced SE produced sustained reductions in spontaneous seizure frequency and cumulative burden and prolonged seizure latency, supporting early regulation of cellular stress pathways as a disease-modifying strategy [38]. However, our interpretation should nevertheless remain cautious because we did not measure the brain concentrations of CDDO-EA, and also equivalent electrographic SE burden before treatment allocation was not formally demonstrated.

Several mechanisms may contribute to this persistent effect, such as SE disrupting the blood–brain barrier, promoting albumin uptake and TGF-β signaling in astrocytes, recruiting peripheral immune cells, and sustaining microglial and cytokine responses that facilitate network reorganization [58,59,60,61,62,63]. Experimental blockade of leukocyte–endothelial adhesion or monocyte recruitment reduces inflammatory injury after seizures, whereas prolonged barrier leakage is associated with progression of temporal lobe epilepsy [58,59,60,61]. Suppression of albumin-TGF-β signaling with losartan can prevent acquired epilepsy in models of blood–brain barrier dysfunction [62,63]. By simultaneously improving antioxidant capacity and reducing inflammatory signaling, CDDO-EA may interrupt several components of this self-reinforcing cascade. Its profile differs from a simple radical scavenger because pharmacological Nrf2 activation can coordinate glutathione metabolism, detoxification, mitochondrial support, and inflammatory regulation [11,13]. This broader response may help explain why treatment restricted to the early post-SE period produced effects that persisted after drug withdrawal.

The present findings can also be considered in the context of previous studies examining pharmacological modulation of glial responses after seizures. The related synthetic triterpenoid CDDO-Me has been reported to inhibit microglial activation and monocyte infiltration following SE, accompanied by attenuation of NF-κB- and p38 MAPK-associated inflammatory signaling [54]. CDDO-Me has also been shown to attenuate reactive astrogliosis and astroglial apoptosis following SE, with effects involving Nrf2-, ERK1/2-, PI3K/AKT-, and NF-κB-related signaling pathways [55]. These findings provide a plausible cellular context for the anti-inflammatory effects of synthetic triterpenoids in epilepsy. However, because the present study measured cytokine mRNA levels in whole-brain homogenates and did not quantify microglial or astrocytic reactivity, we cannot determine whether the effects of CDDO-EA involve direct modulation of either glial population. Future cell-specific studies will be required to establish this relationship.

The chronic findings are also consistent with previous disease-modification studies targeting redox and inflammatory pathways. Pharmacological Keap1 inhibition, combined antioxidant therapy, dimethyl fumarate, selective NOX2 inhibition, thioredoxin-mimetic treatment, and seizure-responsive Nrf2 gene therapy have each reduced one or more measures of epilepsy development or progression [10,12,18,19,20,21]. Such interventions targeting downstream inflammatory mechanisms have likewise shown benefits, including neuronal COX-2 deletion and transient EP2 receptor inhibition, which reduce post-SE inflammation and injury, while silencing miR-134 produces prolonged seizure suppression [64,65,66]. The present study extends this literature by demonstrating that CDDO-EA, a systemically administered synthetic triterpenoid, can influence both acute seizure expression and the later evolution of spontaneous recurrent seizures. Importantly, the combined reduction in seizure frequency, cumulative burden, and delayed seizure onset provides stronger evidence of disease modification than any single endpoint alone.

However, we acknowledge several limitations associated with this study, such as only male rats being included, despite evidence that redox-directed therapies may produce sex-dependent effects after SE [10]. The acute cohort was modest in size, which limited power to detect a difference in convulsive seizure incidence. Moreover, whole-brain homogenates also prevent identification of region- and cell-specific responses, particularly within the hippocampus, cortex, microglia, astrocytes, and neurons.

Although additional Iba1 and GFAP analyses provided information regarding microglial and astrocytic responses during the chronic phase, these measurements were obtained at a single terminal time point and therefore cannot establish the temporal relationship between glial responses, CDDO-EA treatment, and seizure development. In addition, direct indices of Keap1 modification, Nrf2 nuclear translocation, antioxidant-response-element activation, and downstream targets such as NQO1, HO-1, GCLC, and glutathione-associated enzymes were not measured. Pharmacokinetic studies, delayed-treatment paradigms, and genetic or pharmacological disruption of Nrf2 will therefore be necessary to establish target dependency. Dose–response studies will also be required to determine whether the magnitude of the acute anticonvulsant effects of CDDO-EA is dose-dependent and whether higher or repeated exposure produces more robust protection. Because the present study evaluated a single dose, the nonsignificant trends observed for some endpoints cannot be attributed specifically to insufficient dosing. Confirmation of equivalent initial electrographic SE severity, together with longer follow-up, would further strengthen the antiepileptogenic interpretation.

An additional limitation is that non-seizure, untreated animals were not included as a baseline reference group. Although vehicle-treated animals served as the appropriate experimental controls for assessing the effect of CDDO-EA under identical seizure-inducing conditions, inclusion of non-manipulated animals in future studies would allow differentiation between seizure-associated changes and physiological baseline levels.

A further limitation is that behavioral and cognitive outcomes were not assessed in the present study. Although CDDO-EA reduced spontaneous seizure burden and preserved hippocampal neuronal density, these measures do not establish whether treatment also improves, has no effect on, or adversely affects the behavioral and cognitive consequences of acquired epilepsy. Future studies incorporating behavioral and cognitive assessments will be important to determine whether the observed electrophysiological and histological benefits translate into functional improvements.

## 5. Conclusions

In conclusion, the present study supports CDDO-EA as a promising redox- and inflammation-directed therapeutic strategy for acquired epilepsy. CDDO-EA limited progression to severe PTZ-induced convulsions, enhanced antioxidant defenses, and suppressed pro-inflammatory cytokine expression. More importantly, a time-limited intervention initiated after SE delayed spontaneous seizure onset and produced a sustained reduction in seizure progression and cumulative burden. These findings justify further mechanistic and translational evaluation of CDDO-EA as a potential disease-modifying therapy.

## Figures and Tables

**Table 1:** List of primer sequences used for qRT-PCR.

Name	Forward	Reverse	Accession Number
IL-6	GACTTCCAGCCAGTTGCCTT	CTGGTCTGTTGTGGGTGGTAT	NM_012589.2
IL-1β	CCCTGCAGCTGGAGAGTGTGG	AGCACCTCTCAAGCAGAGCACA	NM_031512.2
TNF-α	ATGGGCTCCCTCTCATCAGT	GCTTGGTGGTTTGCTACGA	NM_012675.3
GAPDH	GACATGCCGCCTGGAGAAAC	AGCCCAGGATGCCCTTTAGT	NM_017008.5

## Data Availability

The data presented in this study are available in the article and on request from the corresponding author. The data are not publicly available as there are no additional datasets beyond those presented in the article.
